# Computational model to reproduce fingertip trajectories and arm postures during human three-joint arm movements: minimum muscle-stress-change model

**DOI:** 10.1007/s00422-025-01022-4

**Published:** 2025-08-26

**Authors:** Masazumi Katayama

**Affiliations:** Department of Human and Artificial Intelligent Systems, Graduate School of Engineering, University of Fukui, 3-9-1, Bunkyo, Fukui-shi, Fukui 910-8507 Japan

**Keywords:** Reaching movement, Computational model, Three-joint arm, Muscle stress, Fingertip trajectory, Arm posture

## Abstract

Previous studies on the computational principle for solving the movement selection problem for the human arm have primarily focused on hand trajectories associated with the two-joint movements of the shoulder and elbow joints. Further, only a few computational models, that consider the musculoskeletal system, have been investigated. From this perspective, a minimum muscle-stress-change model was evaluated for the fingertip trajectories and arm postures during three-joint movements in the horizontal plane, including wrist joint rotation. A musculoskeletal model of a three-joint arm with eight muscles was used to perform the optimization calculations that determine the optimal arm movements. Results show that the computational model can reproduce the measured fingertip trajectories and arm postures to an equal or greater extent compared with the minimum angular-jerk model and the minimum torque-change model. Furthermore, the errors of the minimum muscle-stress-change model remained small for different values of joint viscosity, physiological cross-sectional areas, and moment arms, resulting in a small dependency of these parameters. In contrast, the minimum torque-change model resulted in considerable errors under low-viscosity conditions. Consequently, the minimum muscle-stress-change model has emerged as a promising candidate for elucidating the computational principle.

**Supplementary Information:**

The online version contains supplementary material available at 10.1007/s00422-025-01022-4.

## Introduction

Hand trajectories during human reaching movements have an invariant feature. In point-to-point reaching movements in the horizontal plane, with the wrist joint fixed to prevent rotation, the hand trajectory is almost straight; the tangential velocity follows a bell-shaped curve (Abend et al. 1982). This observation suggests that reaching movements are selected based on a common principle of movement selection in the human brain. To elucidate this principle, various computational models have been proposed. The minimum hand-jerk model (HJ) based on the kinematic smoothness constraint of the hand trajectory can reproduce this invariant feature and the hand trajectory through a via point (Flash and Hogan 1985). However, some human hand trajectories are curved during point-to-point reaching movements; the HJ cannot account for these curved hand trajectories because the hand path of the optimal point-to-point movement selected by it is straight. The minimum angular-jerk model (AJ) with the kinematic smoothness constraint of the angular acceleration has a capacity to generate curved hand trajectories (Hogan 1984; Nakano et al. 1999; Wada et al. 2001). The optimal movements selected by the HJ and AJ are not affected by parameters such as the mass of the human arm. The minimum torque-change model (TC) based on the dynamic smoothness constraint of the joint torque for human motor control has been proposed (Uno et al. 1989; Nakano et al. 1999). The optimal movements selected by the TC depend on the human arm’s mass and moment of inertia. The TC can reproduce curved hand trajectories of point-to-point reaching movements and hand trajectories during movements performed using a spring attached to the hand. Notably, computational models that consider the musculoskeletal system of the human arm have also been discussed, including the minimum muscle-tension-change model (Dornay et al. 1996) and the minimum motor-command-change model (Kawato 1996). These computational models may be considered more biologically plausible than the HJ, AJ and TC for the computational process of movement selection in the human brain. However, these computational models have not been evaluated in detail. Moreover, many attractive computational models have been proposed from perspectives different from those based on smoothness constraints. For example, the minimum variance model, which assumes motor command-dependent noise to minimize variation at the end of a movement, can reproduce hand trajectories and eye movements (Harris and Wolpert 1998). A model for generating hand trajectories based on optimal control has also been proposed (Todorov and Jordan 2002). Although this computational model is attractive, it is difficult to explain why a deafferented monkey can perform goal-directed movements (Bizzi et al. 1984). From a different perspective, a minimum expected-muscle-energy-consumption model has been evaluated (Taniai and Nishii 2015).

Regarding the problem of arm posture selection, an infinite number of possible arm postures exist because the human arm has redundant degrees of freedom. Even when the hand is maintained at a certain target position, the arm posture is not uniquely determined. Therefore, it is necessary to select the hand trajectory and arm posture. Although most studies on computational models for movement selection have focused on hand trajectory generation, some others on the arm posture selection problem have focused on investigating reaching movements with the shoulder and elbow joints in three-dimensional space by fixing the wrist joint (Soechting et al. 1995; Wada et al. 2005; Kang et al. 2005; Biess et al. 2007). However, not many studies have been conducted on arm movements with three joints, including the wrist joint. Because redundant degrees of freedom are employed in executing various movements, evaluating the movements of the human three-joint (shoulder, elbow, and wrist joints) arm is also important.

This research involved investigate the generation problem of fingertip trajectory and arm posture by focusing on computational models based on smoothness constraints. From this perspective, a minimum muscle-stress-change model (MSC) was evaluated by measuring the fingertip trajectories and arm postures of the reaching movements of a human three-joint arm in the horizontal plane, including the wrist joint. To assess the computational model’s validity, the AJ and TC, which are appropriate computational models based on smoothness constraints, were also evaluated for comparison.

## Movement selection based on the optimization principle

### Computational models that select fingertip trajectories and arm postures

**Fig. 1:**
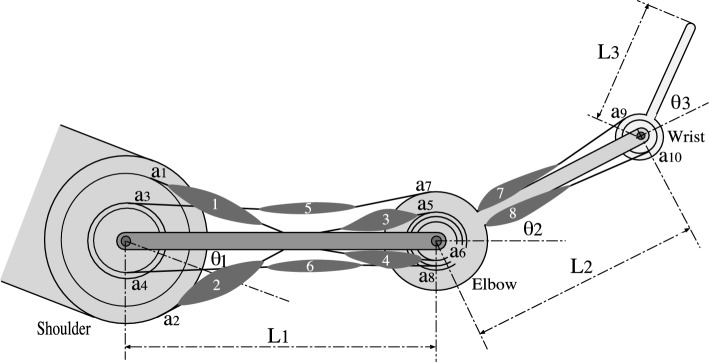
Musculoskeletal model of a three-joint arm with eight muscles

The activities of the multiple muscles involved in performing a hand-force maintenance task can be explained by the muscle tensions calculated by minimizing the sum of the square or cube of the muscle stresses, with the former being more accurate (Crowninshield and Brand 1981; van Bolhuis and Gielen 1999; Gomi 2001). This finding suggests that an MSC based on a smoothness constraint of muscle stress may reproduce human arm movements. This computational model selects optimal arm movements that minimize the following evaluation value $C_{MSC}$.

1 $$\begin{aligned} C_{MSC} = \frac{1}{2}\int _0^{tf} \sum _{i=1}^m \left( \frac{d S_{i}}{dt} \right) ^2 dt, ~~~ S_i = \frac{T_i}{PCSA_i}, \end{aligned}$$

where $S_i$ denotes the *i* th muscle stress and is calculated by dividing the muscle tension $T_i$ by the physiological cross-sectional area $PCSA_i$ of the *i* th muscle. $t_f$ represents the movement time required to move from the initial position to the final one. The value of *m* was 8 because the musculoskeletal model shown in Fig. 1 was modeled with eight muscles.

The optimal movements of the MSC were determined through two-step optimization. The first optimization step involved selecting the optimal muscle tension $\mathbf{T}$ at time *t* by minimizing the involved evaluation value *E* under the condition that constraints regarding the joint torque and muscle tension were satisfied:

2 $$\begin{aligned} E = \sum _{i=1}^{m} {S_i}^2, ~~~ \text {subject~to}~~~ \varvec{\tau } = \mathbf{A}\mathbf{T}, ~~ {T_i \ge 0} \end{aligned}$$

where the joint torque $\varvec{\tau }$ is generated by the product of the muscle tension ${\mathbf{T}}$ and moment arm ${\mathbf{A}}$. This objective function is the same as that reported by Crowninshield and Brand (1981), van Bolhuis and Gielen (1999), and Gomi (2001). In the second optimization step, the MSC selects optimal fingertip trajectories and arm postures during movement by minimizing $C_{MSC}$. Herein, it is noted that this two-step optimization does not guarantee that the global solution calculated by minimizing only the evaluation value $C_{MSC}$ of Eq. 1 can be obtained.

Although the AJ and TC can determine optimal fingertip trajectories and arm postures, their capability for the three-joint arm is yet to be investigated. Herein, the AJ and TC were also evaluated for comparison with the MSC. However, the HJ was not included in the evaluation due to its inability to determine arm postures. The AJ selects the optimal arm movements that minimize the following evaluation value $C_{AJ}$:

3 $$\begin{aligned} C_{AJ} = \frac{1}{2}\int _0^{tf} \sum _{i=1}^n \left( \frac{d^3 \theta _i}{dt^3} \right) ^2 dt, \end{aligned}$$

where *n* = 3 for a three-joint arm, $\theta _i$ represents the joint angle of the *i* th joint, and $t_f$ is the movement time. The TC selects the optimal movements that minimize the following evaluation value $C_{TC}$:

4 $$\begin{aligned} C_{TC} = \frac{1}{2}\int _0^{tf} \sum _{i=1}^n \left( \frac{d \tau _{i}}{dt} \right) ^2 dt, \end{aligned}$$

where $\tau _{i}$ is the joint torque of the *i* th joint.

Optimal arm postures and fingertip trajectories of the computational models were determined herein using the real-coded genetic algorithm that was developed to deal with multiple local minima and easily implement computational models of various objective functions (see Supplementary Material for details). In a general genetic algorithm, individuals are represented as binary numbers. However, for the movement selection problem, it is necessary to consider many types of constraints, such as the achievability of fingertip positions, the possibility of arm postures, and the smoothness of an arm movement. In such a problem, using a real-coded genetic algorithm with individuals that use real numbers is advantageous. For this algorithm, time series data for each axis (x-axis and y-axis) of the fingertip trajectory and wrist joint angle was divided into six equal sections, such that the number of points for each is seven, resulting in a seven-dimensional vector, similar to the optimization method of Harris and Wolpert (1998). Therefore, each individual was expressed by three vectors with seven dimensions: two vectors that determined the x- and y-axis coordinates of the fingertip trajectory and one vector that determined the wrist joint angles to determine the arm postures during the movement. The initial and final elements of the x- and y-coordinate vectors were set to the initial and final fingertip positions of the measured arm movement, respectively. Similarly, the first element of the wrist joint angle vector was set to the wrist joint angle of the measured arm posture at the initial position. However, the final element was not specified, and the final arm posture was selected through optimization. In this real-coded genetic algorithm, first, seven points of each seven-dimensional vector of each individual were smoothly interpolated using quintic spline interpolation to generate time series data. Next, the joint angle was calculated from the generated time series data using the kinematics equation for a three-joint arm. Then, the joint torque was calculated using the dynamics equation. In the first optimization step of the MSC, the muscle tension that minimized the evaluation value *E* of the objective function in Eq. 2 was determined from the joint torque. This optimization is possible using quadratic programming, a well-known constrained optimization method. The muscle stress, calculated from the muscle tension, was substituted into the objective function in Eq. 1. By repeating this process from the start time to the end time of the movement, the evaluation value $C_{MSC}$ of each individual was calculated. The next generation of individuals was selected from all individuals. This process was repeated to find an approximation of the optimal solution in the second optimization step. In the AJ and TC, the evaluation value of the objective function can be calculated using the obtained joint angle and joint torque, respectively. Because the solution is likely dependent on the initial individuals, optimization was repeated thrice under the same condition; the optimal solution was selected. Notably, the three solutions were found to be nearly identical. In addition, we confirmed that the developed algorithm can accurately determine the optimal two-joint reaching movements of the HJ and AJ (see Supplementary Material).

### Musculoskeletal model of the three-joint arm

The dynamics equation for a three-joint arm in the horizontal plane is as follows (see Supplementary Material):

5 $$\begin{aligned} \mathbf{M}(\varvec{\theta }) \ddot{\varvec{\theta }} + \mathbf{H}(\varvec{\theta },\varvec{\dot{\theta }}) + \mathbf{D} \dot{\varvec{\theta }} = {\varvec{\tau }}, \end{aligned}$$

where $\varvec{\tau }= (\tau _1, \tau _2, \tau _3)^T$, $\varvec{\theta } = (\theta _1, \theta _2, \theta _3)^T$, $\varvec{\dot{\theta }} = (\dot{\theta }_1, \dot{\theta }_2, \dot{\theta }_3)^T$ and $\varvec{\ddot{\theta }} = (\ddot{\theta }_1, \ddot{\theta }_2, \ddot{\theta }_3)^T$ denote vectors of the joint torque, joint angle, angular velocity, and angular acceleration, respectively. The matrices $\mathbf{M}$ and $\mathbf{D}$ represent the inertia and joint viscosity, respectively. $\mathbf{H}$ is a vector of the Coriolis and centrifugal forces.

The joint torque $\varvec{\tau }$ is generated by the product of muscle tension ${\mathbf{T}}$ and moment arm ${\mathbf{A}}$.

6 $$\begin{aligned} \varvec{\tau } = \mathbf{A}\mathbf{T}, \end{aligned}$$

where

7 $$\begin{aligned} \mathbf{T}&= \left( T_1, T_2, T_3, T_4, T_5, T_6, T_7, T_8\right) ^T , \end{aligned}$$

8 $$\begin{aligned} \mathbf{A}&= \left( \begin{array}{cccccccc} a_1 & a_2 & 0 & 0 & a_3 & a_4 & 0 & 0 \\ 0 & 0 & a_5 & a_6 & a_7 & a_8 & 0 & 0 \\ 0 & 0 & 0 & 0 & 0 & 0 & a_9 & a_{10} \end{array}\right) . \end{aligned}$$

Here, $$T_i$$ denotes the tension of the *i* th muscle. $$T_1$$ and $$T_2$$, $$T_3 $$ and $$ T_4$$, $$T_5$$ and $$T_6 $$, and $$T_7$$ and $$T_8$$ represent the muscle tensions of the flexor and extensor of the shoulder joint, respectively, the flexor and extensor of the elbow joint, respectively, the biarticular flexor and extensor of the shoulder and elbow joints, respectively, and the flexor and extensor of the wrist joint, respectively (Fig. 1). $$a_i$$ denotes the moment arm for each muscle.

The elements of the joint viscosity $\mathbf{D}$ are expressed as follows:

9 $$\begin{aligned} \mathbf{D} = \left[ \begin{array}{ccc} D_{11} & D_{12} & 0 \\ D_{21} & D_{22} & 0 \\ 0 & 0 & D_{33} \end{array} \right] . \end{aligned}$$

Here, the diagonal components $D_{11}$, $D_{22}$, and $D_{33}$ represent the viscous coefficients at the shoulder, elbow, and wrist joints, respectively, with $D_{12}$ and $D_{21}$ being the coefficients of the interaction between the shoulder and elbow joints. Herein, the values of $D_{11}$, $D_{21}$, $D_{12}$, and $D_{22}$ were determined using quantitative data obtained during human arm movements (Gomi and Kawato 1995). These values depend on the average values of the joint torques during movement (Nakano et al. 1999). The value of $D_{33}$ for the wrist joint was set to the average value of the shoulder and elbow joints as a reference.

10 $$\begin{aligned} {\begin{matrix} D_{11} = 0.63\alpha _A + 0.095 |\bar{\tau }_1|, \\ D_{21} = D_{12} = 0.175\alpha _A + 0.0375 |\bar{\tau }_2|, \\ D_{22} = 0.76\alpha _A + 0.185 |\bar{\tau }_2|, \\ D_{33} = 0.695 \alpha _A \alpha _B + 0.140 |\bar{\tau }_3|, \end{matrix}} \end{aligned}$$

where $\bar{\tau }_1$, $\bar{\tau }_2$, and $\bar{\tau }_3$ represent the average torques of the shoulder, elbow, and wrist joints, respectively. $\alpha _A$ and $\alpha _B$ were used to examine the effect of joint viscosity on optimal arm movements. $\alpha _B$ modulated only the value of $D_{33}$ in the wrist joint, and $\alpha _A$ influenced the viscosity across all the joints. Table 1 lists the abbreviations corresponding to the coefficients used to determine the viscosities of the three joints.

**Table 1:** Scale factors that determine the viscosity values of the three joints

	B05A10	B10A10	B20A10	B10A05	B10A20
$\alpha _A$	1.0	1.0	1.0	0.5	2.0
$\alpha _B$	0.5	1.0	2.0	1.0	1.0

It is necessary to use the physical parameters of the participant’s arm to perform the optimization calculation for movement selection. In the AJ with the kinematic smoothness constraint, only the link lengths of the upper arm, forearm, and hand are required. Each participant’s lengths were directly measured. In the TC and MSC with the dynamic smoothness constraint, in addition to the link lengths, the following parameters of Eq. 5 are required: mass, center of mass, and moment of inertia of each link. Herein, we developed a method for estimating precise values of these parameters for each participant (Appendix A). In this method, the upper arm and forearm were modeled using three cylinders each, whereas the hand was modeled using two plates. The volumes of these cylinders or plates corresponding to each participant’s arm were calculated, and the mass of each link was estimated using the volume ratios and specific gravity of the fat, muscle, and bone. Table 2 lists the estimated physical parameters of the participants’ arms. Further, the MSC requires the physiological cross-sectional area (PCSA) and moment arm of each muscle, in addition to a musculoskeletal model of the human arm. However, because it is difficult to estimate the true values of the joint viscosity, PCSAs, and moment arms for each subject, we examined how the optimal arm movements of each computational model depend on these parameters instead of estimating their true values. While, although researchers have studied the muscles involved in the horizontal flexion and extension of the shoulder and elbow joints, the number and types of muscles involved in these movements considerably vary. Herein, the MSC was evaluated based on the values for eight combinations of PCSAs and moment arms (Tables 3 and 4). The muscles selected in S11, S12, S21, and S22 were determined by Ito and Takano (2012), and those in S31, S32, S41, and S42 were determined by Nakamura and Saito (1992) (see Supplementary Material). S21, S22, S41, and S42 include the supplementary muscles. The PCSA values of the muscles of the shoulder and elbow joints were obtained from Meek et al. (1990), and the values around the wrist joint were obtained from Gonzalez et al. (1997). The PCSA value of each muscle in the musculoskeletal model was calculated as the sum of the PCSA values of the muscles playing the same role in each muscle selection. The moment arms of the shoulder and elbow joints for the muscle selections of S11, S21, S31, and S41 were obtained from Meek et al. (1990), and those in the wrist joint were determined from Gonzalez et al. (1997). The moment arms of S12, S22, S32, and S42 in the shoulder joint were obtained from Kuechle et al. (1997), those in the elbow joint from Meek et al. (1990), and those in the wrist joint from Gonzalez et al. (1997). The moment arm of each muscle in the musculoskeletal model was the average of those of the muscles playing the same role in each muscle selection.

**Table 2:** Parameter values measured or estimated from the arms of the participants

$L_1$ (m)	$L_2$ (m)	$L_3$ (m)	$L_{g1}$ (m)	$L_{g2}$ (m)	$L_{g3}$ (m)
0.283	0.240	0.195	0.110	0.0928	0.0674
±0.017	±0.009	±0.011	±0.007	±0.0048	±0.0024

$m_1$ (kg)	$m_2$ (kg)	$m_3$ (kg)	$I_1$ ($\hbox {kgm}^2$)	$I_2$ ($\hbox {kgm}^2$)	$I_3$ ($\hbox {kgm}^2$)
1.617	0.878	0.512	0.0363	0.0128	0.00438
±0.201	±0.092	±0.055	±0.0064	±0.0018	±0.00067

The upper value is the mean of each participant, and the lower value is the standard deviation (SD) across participants. $L_i$, $L_{gi}$ and $m_i$ correspond to the link length, center of mass, and mass of the *i* th link, respectively, and $I_i$ denotes the moment of inertia around the *i* th joint. Links 1, 2, and 3 correspond to the upper arm, forearm, and hand, respectively

**Table 3:** Physiological cross-sectional areas (PCSAs) of the eight muscles

Muscle	SF	SE	EF	EE	DF	DE	WF	WE
selection								
S11 and S12	10.97	3.87	13.40	9.74	3.23	3.87	6.1	6.8
S21 and S22	10.97	16.77	14.69	9.74	3.23	3.87	6.1	6.8
S31 and S32	24.52	25.80	9.03	7.74	3.23	3.87	6.1	6.8
S41 and S42	24.52	44.52	14.69	9.74	3.23	3.87	6.1	6.8

SF and SE, EF and EE, DF and DE, and WF and WE denote the monoarticular flexors and extensors of the shoulder and elbow joints, respectively, the biarticular flexors and extensors of the shoulder and elbow joints, respectively, and the monoarticular flexor and extensor of the wrist joint, respectively. The PCSA values are expressed in $\hbox {cm}^2$

**Table 4:** Moment arms of the eight muscles

	$a_1$	$a_2$	$a_3$	$a_4$	$a_5$	$a_6$	$a_7$	$a_8$	$a_9$	$a_{10}$
S11	4.06	$-$7.87	2.92	$-$2.54	1.98	$-$1.68	4.32	$-$2.29	1.70	$-$1.07
S12	2.84	$-$2.46	2.92	$-$2.54	1.98	$-$1.68	4.32	$-$2.29	1.70	$-$1.07
S21	4.06	$-$5.97	2.92	$-$2.54	2.42	$-$1.68	4.32	$-$2.29	1.70	$-$1.07
S22	2.84	$-$1.40	2.92	$-$2.54	2.42	$-$1.68	4.32	$-$2.29	1.70	$-$1.07
S31	4.57	$-$3.12	2.92	$-$2.54	2.67	$-$2.03	4.32	$-$2.29	1.70	$-$1.07
S32	2.88	$-$1.57	2.92	$-$2.54	2.67	$-$2.03	4.32	$-$2.29	1.70	$-$1.07
S41	4.57	$-$3.53	2.92	$-$2.54	2.42	$-$1.68	4.32	$-$2.29	1.70	$-$1.07
S42	2.88	$-$1.16	2.92	$-$2.54	2.42	$-$1.68	4.32	$-$2.29	1.70	$-$1.07

Positive and negative values represent the flexors and extensors, respectively. The values of the moment arms are given in cm

**Fig. 2:**
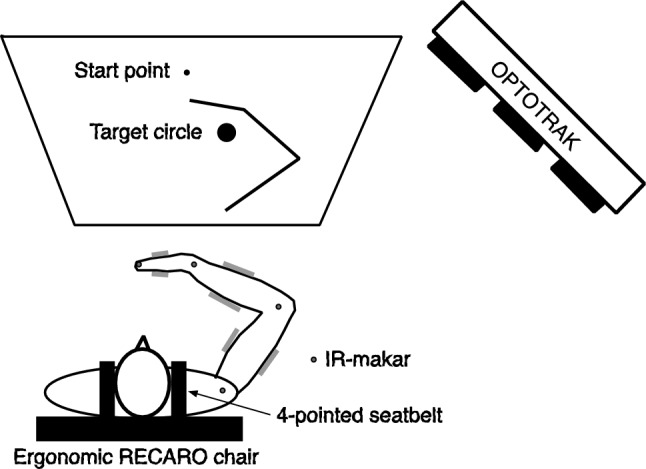
Experimental setup. The start point and target circle were presented on a screen in front of the participant, as well as a stick picture of the arm posture. The participant was seated in an ergonomically designed RECARO chair and securely fastened with a four-point racing harness. Four infrared markers were attached to the shoulder, elbow, wrist, and fingertip to record arm movements

**Fig. 3:**
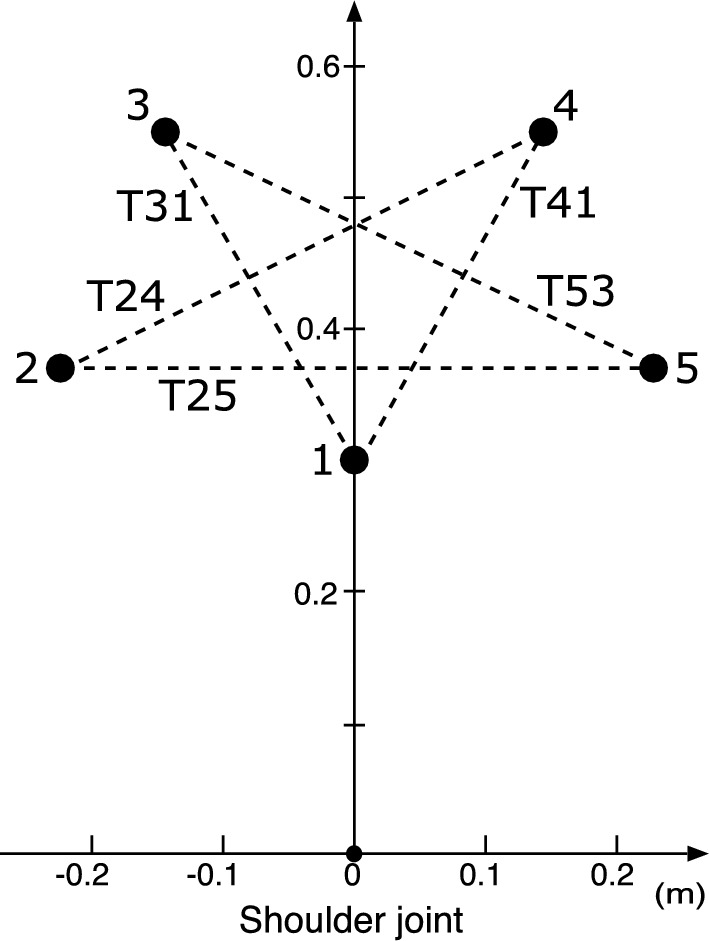
Start and target points for arm movements. T24, T25, T31, T41, and T53 denote movements from 2 to 4, from 2 to 5, from 3 to 1, from 4 to 1, and from 5 to 3, respectively. The x- and y-axis coordinates of these points were (0.0,0.3), ($-$0.21,0.37), ($-$0.15,0.56), (0.15,0.56), and (0.21,0.37), respectively. The origin of the coordinates is defined as the center of rotation of the shoulder joint

**Table 5:** Movement times of the movement directions

Initial wrist angle	T24	T25	T31	T41	T53
$0^\circ$	0.672	0.663	0.550	0.537	0.686
	±0.023	±0.034	±0.017	±0.028	±0.017
$30^\circ$	0.658	0.640	0.551	0.530	0.654
	±0.034	±0.023	±0.012	± 0.028	±0.033

The upper value is the mean value of each participant, with the lower value being the SD

## Measurement experiment of arm movements

Five right-handed healthy adults (mean age: 21.4 ± 0.9 years, mean height 1.69 ± 0.09 m; four males and one female) participated in this measurement experiment. All the participants had normal or corrected-to-normal vision. They were naïve to the purpose of this study and provided written informed consent to participate. They received book coupons as a token of appreciation for their participation. The experimental protocol was approved by the Bioethics Committee of Human and Artificial Intelligent Systems at University of Fukui (#H181201).

Figure 2 shows an experimental environment to measure the fingertip trajectories and arm postures during reaching movements. The participants were seated in an ergonomically designed RECARO chair to reduce performance loss due to fatigue. They were also secured with four-point racing seatbelts to prevent displacement of the shoulder joint position. To eliminate muscle activities that support the arm against gravity, the hand, forearm, and upper arm of each participant were supported by a thin fishing line (PE 0.8) from a 4-m-high ceiling. To capture their arm movements, four infrared markers were placed at the tip of the index finger, and each center of rotation of the shoulder joint, elbow joint, and wrist joint, and measurements were performed at 200 Hz using the OPTOTRAK3020 system (Northern Digital Inc., Waterloo, Canada). The position data from the four markers were digitally filtered in both directions using a fourth-order Butterworth filter with a cutoff frequency of 10 Hz. Movement onset and offset were determined using the two-dimensional curvature values, which typically exhibit sharp increases near these points (e.g., Nakano et al. 1999). The start time was identified by searching backward in time from the movement midpoint to detect the first point where the curvature exceeded a threshold of 0.3 $\hbox {mm}^{-1}$, while the end time was determined by searching forward from the midpoint using the same threshold. This curvature-based method enabled more accurate and robust detection, with fewer failures, than a method based on tangential velocity. Outliers in the measured data were subsequently removed.

A screen was placed in front of the participant, on which the start point and target circle were displayed. The target circle was delineated with a larger radius than the start point to avoid requiring excessive precision in movement. In addition, a stick picture was displayed to show the fingertip position and arm posture during the movement (Fig. 2). The arm movements were measured in five types of movement directions: T24, T25, T31, T41, and T53 (Fig. 3). First, the participants freely moved their arms for 3–5 min to familiarize themselves with the experimental environment. They then practiced performing the movement task within the instructed time. The movement time ranged for T24, T25, and T53 is 600±50 ms, and that for T31 and T41 is 500±50 ms. If the movement time was outside the range, the participant was instructed to "please move a little faster" or "please move a little slower." In the next training session, three types of exercises were performed: (1) those with no wrist joint rotation, (2) those with intentional wrist joint rotation, and (3) those with natural movements without instruction. They practiced the three types of exercises in three trials each (nine trials in total). Next, the participants repeatedly practiced the movement task, starting with the instructed initial wrist angle. The initial wrist angles were $0^\circ$ and $30^\circ$. The number of trials was adjusted according to the performance levels of the participants. After the training session, arm movements were measured for 30 trials in each movement direction, with an initial wrist angle of $0^\circ$ (150 trials in total). The movement directions were randomized for each trial. Arm movements with an initial wrist angle of $30^\circ$ were subsequently measured using the same procedure.

In each trial, the participants were instructed to make a slight shaking motion of the hand near the start point after the first beep, similar to a golf waggle, to allow them to relax as much as possible. The participant then placed the fingertip at the start point with the fingers slightly extended while maintaining the arm posture at the specified initial wrist angle. The wrist angle was displayed on the screen, and the participant adjusted it to the specified initial wrist angle while looking at the screen. After the second beep, the participant moved the arm such that the fingertip entered the target circle and maintained the arm posture until the third beep.

**Fig. 4:**
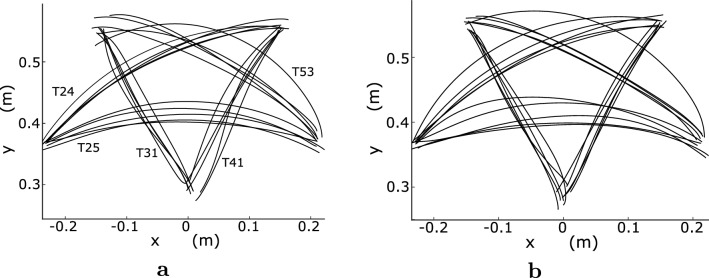
Measured fingertip paths for all participants, with **a** and **b** showing the results of the initial wrist angles of $0^\circ$ and $30^\circ$, respectively

**Fig. 5:**
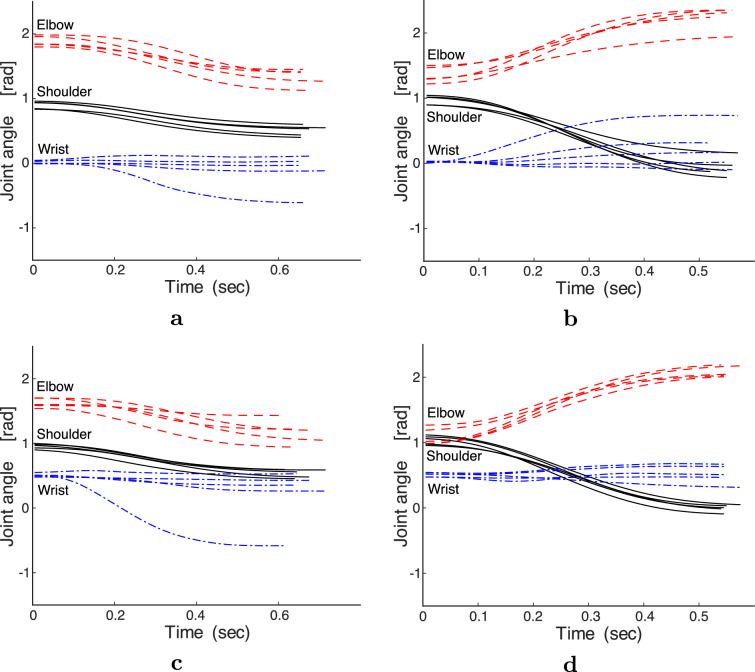
Measured joint angles for all participants in each condition. **a** T24, $0^\circ$. **b** T31, $0^\circ$. **c** T24, $30^\circ$. **d** T31, $30^\circ$

## Results

### Measured fingertip trajectories and arm postures

The observed movement times were largely within the specified range owing to the instructions (Table 5). However, some movement times were slightly long because there were no instructions for the movement time before the movement trial. Although the movement time varied according to the movement direction, the interparticipant variance was small in the same direction. Figure 4 shows that at both initial wrist angles, the fingertip paths at T31 and T41 were approximately straight, whereas those at T24, T25, and T53 were relatively curved. The fingertip paths between the participants varied more when they were curved than when they were straight. The wrist joint was rotated at T31 at an initial wrist angle of $$0^\circ $$, whereas it exhibited little rotation at T24, with one exception, as shown in Fig. 5. At T24 and T31 of $$30^\circ $$, the wrist joint was rotated slightly. For initial wrist angles of $$0^\circ $$ and $$30^\circ $$ in other movement directions, the wrist joint was slightly rotated at T53, and the wrist joints at T25 and T41 were rotated more than the wrist joints at T31 and T53 of $$30^\circ $$. When the rotation angle of the wrist joint was small, there was negligible variation among the participants, especially at T53 of $$0^\circ $$ and T31 and T53 of $$30^\circ $$. As the rotation angle of the wrist joint increased, the variation in the arm postures increased. In addition, the waveform of the wrist joint angle at $$30^\circ $$ resembles that at $$0^\circ $$ shifted upward because the waveform at $$30^\circ $$ is observed to be 0.2$$-$$0.5 rad ($$11.5^\circ $$ $$-$$$$28.6^\circ $$) larger than that at $$0^\circ $$.

**Fig. 6:**
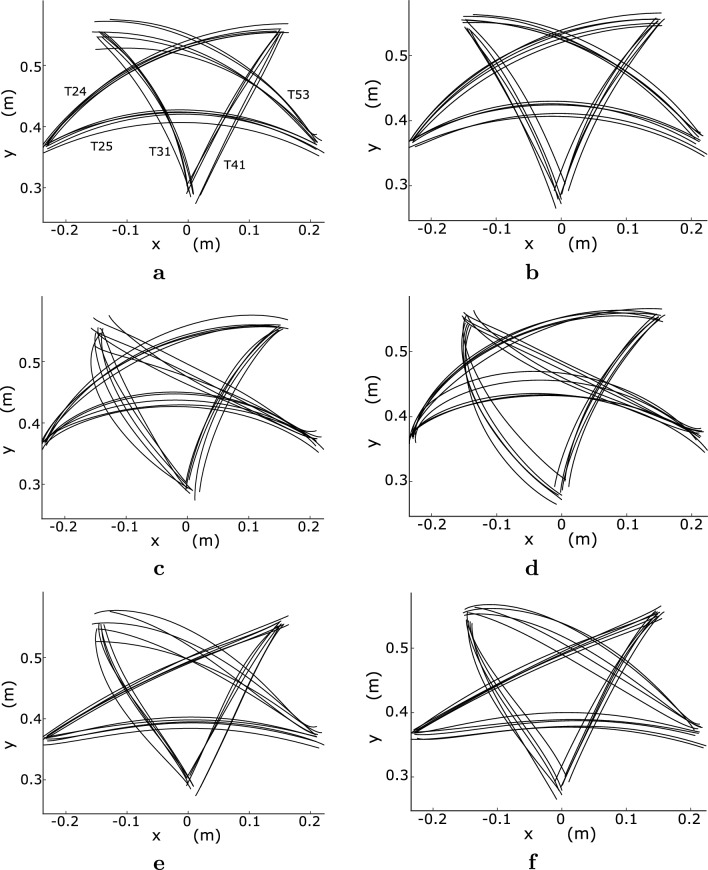
Optimal fingertip paths for each computational model. **a** AJ, $0^\circ$. **b** AJ, $30^\circ$. **c** TC, $0^\circ$. **d** TC, $30^\circ$. **e** MSC, $0^\circ$. **f** MSC, $30^\circ$. (viscosity condition: B10A10 and muscle selection: S21)

**Fig. 7:**
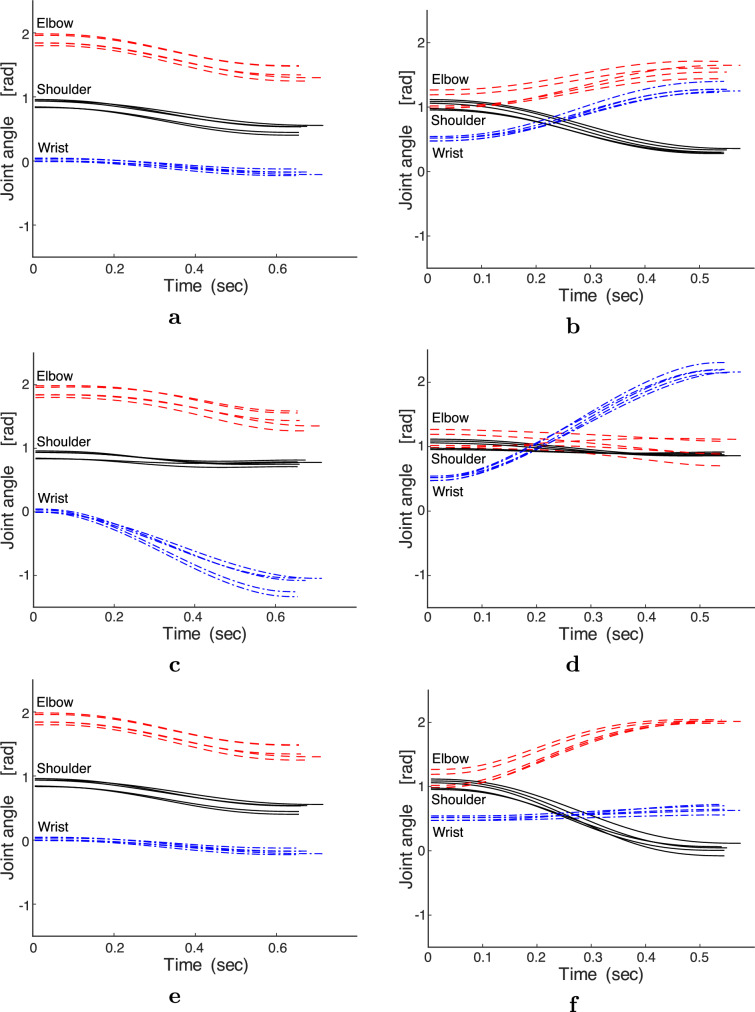
Examples of optimal joint angles of all participants. **a** AJ, T24, $0^\circ$. **b** AJ, T31, $30^\circ$. **c** TC, T24, $0^\circ$. **d** TC, T31, $30^\circ$. **e** MSC, T24, $0^\circ$. **f** MSC, T31, $30^\circ$. (viscosity condition: B10A10 and muscle selection: S21)

**Fig. 8:**
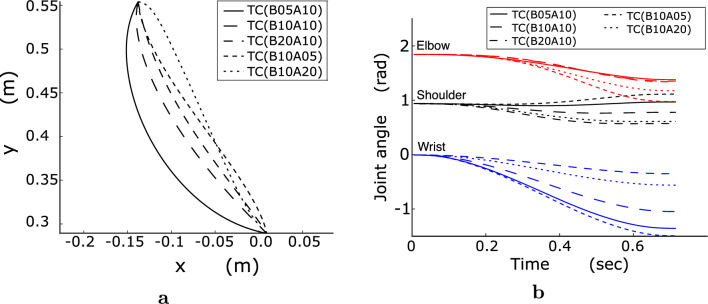
Typical examples of viscous dependency of TC in the optimal fingertip paths and joint angles. **a** Fingertip paths of T31. **b** Joint angles of T24. (initial wrist angle: $0^\circ$ and muscle selection: S21)

**Fig. 9:**
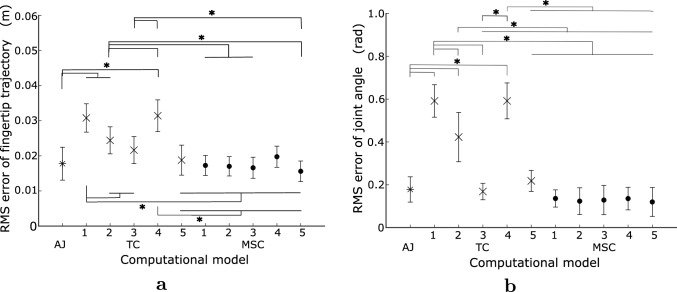
RMS error of optimal arm movements, including the results of $0^\circ$ and $30^\circ$ of the initial wrist angle. **a** Fingertip trajectory and **b** Arm posture. The viscosity conditions 1, 2, 3, 4, and 5 correspond to B05A10, B10A10, B20A10, B10A05, and B10A20, respectively. The vertical lines represent the SD between the RMS errors for the movement directions of all participants. (muscle selection: S21. *$p < 0.05$)

**Fig. 10:**
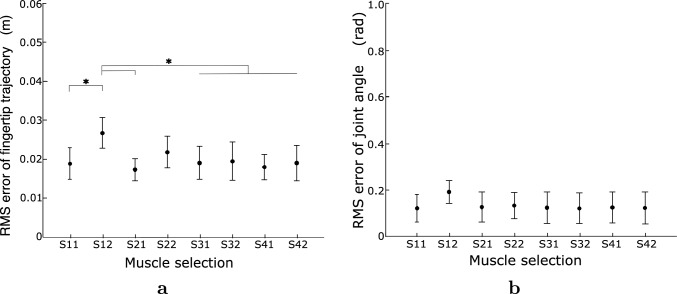
RMS error for each muscle selection, including the results of $0^\circ$ and $30^\circ$ of the initial wrist angle. **a** Fingertip trajectory and **b** Arm posture. The vertical lines represent the SD between the RMS errors for the movement directions of all participants. (computational model: MSC and viscosity: B10A10. *$p < 0.05$)

### Optimal fingertip trajectories and arm postures

As illustrated in Fig. 6, the optimal fingertip paths were similar between initial wrist angles of $0^\circ$ and $30^\circ$ for each computational model; however, they differed among the computational models. Under the viscosity condition of B10A10, the fingertip paths of the AJ and TC for T31 were oppositely curved, whereas those of the MSC were approximately straight. The optimal fingertip paths of T41 were almost straight, with non-significant differences among the three computational models. For T53, the optimal fingertip paths of the AJ and MSC were curved, whereas those of the TC were straight. For T24, the optimal fingertip paths of the AJ and TC were curved while those of the MSC were almost straight. Figure 7 presents the variation in the joint angle of the optimal movement over time for each participant in each computational model. For both initial wrist angles, the rotation angle of the wrist joint was the smallest in the MSC, and the wrist joint of the AJ rotated slightly more than in the MSC but significantly less than in the TC. In the TC, the rotation angle of the wrist joint was significantly larger than that of the AJ and MSC. From a theoretical perspective, the optimal fingertip paths and arm postures in the TC and MSC are dependent on joint viscosity. The TC was strongly dependent on joint viscosity, as shown in Fig. 8. As the joint viscosity decreased, the curvature of the fingertip path and rotation angle of the wrist joint both increased. However, as described below, the MSC was largely independent of joint viscosity.

The root mean square (RMS) errors of the optimal fingertip trajectories and joint angles for each computational model at each joint viscosity are presented in Fig. 9. Because errors at initial wrist angles of $0^\circ$ and $30^\circ$ showed the same tendency, the results included errors for $0^\circ$ and $30^\circ$. The RMS errors were statistically evaluated using the Tukey-Kramer test under all conditions. The fingertip trajectory errors were small for the AJ, for all viscosity conditions of the MSC, and for high-viscosity conditions of the TC. For the TC, the error increased considerably with decreasing wrist joint viscosity. Conversely, the MSC exhibits little viscosity dependency because its errors are small for all viscosity conditions. The tendency of RMS errors of the joint angles shown in Fig. 9b is similar to that of the RMS error of the fingertip trajectories. In the low-viscosity condition for the TC, the errors were numerous because the wrist joint rotated excessively. Figure 10 presents the RMS errors for the MSC calculated using eight combinations of PCSAs and moment arms. The errors in S12 of Fig. 10a, b were slightly larger than those of the other muscle selections; the differences for the fingertip trajectories were statistically significant ($p < 0.05$) and those for the joint angles were not statistically significant ($p > 0.05$). The MSC’s optimal movement should be strongly affected by the PCSAs and moment arms because muscle stress is incorporated into the objective function. Nevertheless, the MSC’s error was little affected by the PCSAs and moment arms, especially with regard to arm posture.

**Fig. 11:**
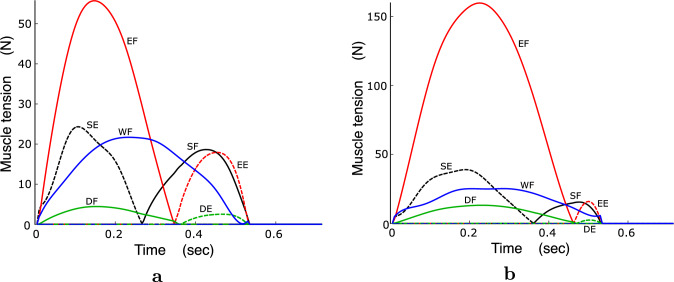
Examples of the muscle tensions for MSC. **a** B10A05. **b** B10A20 (muscle selection: S21, movement direction: T41, solid line: flexor, and dashed line: extensor)

### Muscle tensions and muscle stresses in the optimal movements

Figure 11 shows the muscle tensions for B10A05 and B10A20. In B10A05, which is a low-viscosity condition, the agonist muscles are active during the initial phase of the movement, and the antagonist muscles become active during the subsequent phase. In B10A20, which is a high-viscosity condition, the agonist muscles are primarily active throughout the movement, whereas the activation of the antagonist muscles are small. This is because the agonist muscles must be activated to counteract the viscous torque.

**Fig. 12:**
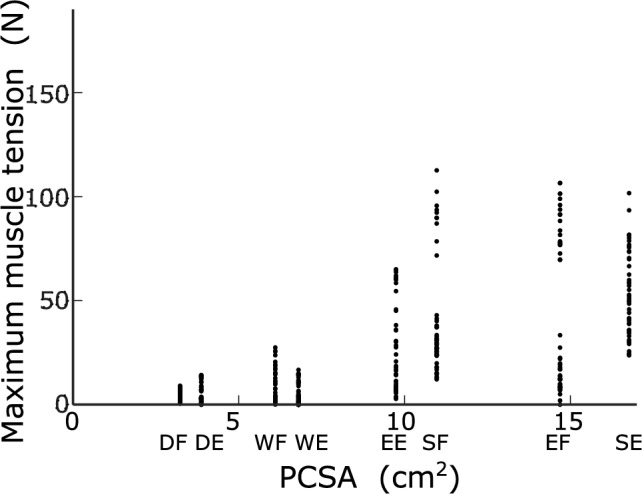
Relation between the maximum muscle tension and the PCSA of each muscle (muscle selection: S21 and viscosity: B10A10)

To examine the relation between muscle tension and PCSA for each muscle, the relation between the maximum muscle tension in each movement and the PCSA for each muscle is presented in Fig. 12. With respect to the muscle tensions in the optimal movement selected by the MSC, the smaller the PCSA of the muscle (i.e., the thinner the muscle), the smaller the selected muscle tension. This indicates that the load on a thin muscle was low. Conversely, the thicker the muscle, the higher the selected muscle tension, indicating that thicker muscles contribute more considerably to movement.

## Discussion

### Joint viscosity

The plausibility of the TC should be evaluated using the true values of joint viscosity during movement because when using the high joint viscosity values of B20A10 and B10A20, the TC can reproduce human arm movement to the same extent as the AJ and MSC. Therefore, it is important to discuss whether it is appropriate to examine the five types of joint viscosity presented in Table 1. Numerous studies have measured joint viscosity at the wrist and/or elbow; however, the reported values vary considerably, even during unconstrained reaching movements that do not involve actions such as grasping (e.g., Hayes and Hatze 1977; Bennett et al. 1992; Sinkjær and Hayashi 1989; Milner and Cloutier 1998; Gomi and Kawato 1995). These discrepancies are primarily attributable to differences in the methods used to apply mechanical perturbations, the estimation procedures, and the level of coactivation between agonist and antagonist muscles. Accordingly, such measurements require careful methodological consideration. However, many previous studies did not adequately consider these factors. In contrast, Gomi and Kawato (1995) addressed these methodological issues by refining the perturbation and estimation methods. They also developed a manipulandum that allowed for arm movement measurements while slightly lifting the participant’s arm above the experimental table using air pressure, minimizing gravitational effects and muscle coactivation. This method enabled the simultaneous and accurate estimation of both the diagonal ($D_{11}$ and $D_{22}$ in Eq. 9) and off-diagonal ($D_{12}$ and $D_{21}$) elements of the joint viscosity matrix. From this perspective, the element values under the B10A10 condition in Table 1 were determined using the estimated values reported by Gomi and Kawato (1995). In this study, to reduce gravitational effects and muscle coactivation, the participant’s arm was suspended from the ceiling. Additionally, the participants performed three types of preparatory exercises along with a golf-waggle-like motion at the initial position. Based on these considerations and in view of the inter-individual variability in joint viscosity, it is appropriate to evaluate the plausibility of the computational models using the five different viscosity conditions listed in Table 1.

### Evaluation of computational models for arm movement selection

In this study, the first optimization step of the MSC is proposed based on the findings of previous studies in which the muscle tensions obtained by minimizing the evaluation value *E* of Eq. 2 reproduce the electromyogram measured when performing a hand-force maintenance task. Therefore, the optimization of the first optimization step is plausible from a physiological perspective. Furthermore, computational models such as the TC and MTC are based on smoothness constraints (time derivative of the parameter) of parameters related to movement control. Therefore, Eq. 1 based on the time derivative of muscle stress is considered the most promising candidate as an objective function in the second optimization step.

The characteristic that the optimal arm movements of the MSC are independent of joint viscosity may be advantageous. When joint viscosity changes due to the coactivation of agonist and antagonist muscles, the optimal arm movement of the TC significantly changes, and the arm movement must be replanned according to joint viscosity. However, because the optimal arm movements of the MSC are hardly affected by joint viscosity, precise movement is possible even without replanning if only minor changes occur when combined with feedback control. Therefore, the challenge of variations in viscosity due to coactivation is not a downside of MSC. On the other hand, such problems do not arise in the AJ, as joint viscosity is not computationally involved. From a theoretical perspective based on the MSC’s objective function, the optimal arm movements should be significantly affected by not only the PCSA but also the moment arm. However, this was not the case in this study. Although this is a critical feature for motor planning and control, it is an open question whether it is advantageous for the MSC. However, it may be advantageous for the MSC that arm movements do not need to be replanned even when the muscles hypertrophy through strength training.

To the best of our knowledge, most previous studies on computational models for movement selection focused on two-joint reaching movements of the shoulder and elbow joints in the horizontal and vertical planes (Flash and Hogan 1985; Uno et al. 1989; Nakano et al. 1999) and three-dimensional space (Soechting et al. 1995; Wada et al. 2005; Kang et al. 2005; Biess et al. 2007). In this study, I focused on three-joint arm movements, (which have rarely been evaluated), and evaluated the performance of each computational model. I found that the AJ and MSC could properly reproduce human three-joint reaching movements. Furthermore, this study found features that could not be obtained in studies of two-joint arm movements (e.g., dependency on joint viscosity and the performance of the TC). The TC can reproduce the hand trajectories for human two-joint reaching movements of the shoulder and elbow joints (Uno et al. 1989; Nakano et al. 1999; Wada et al. 2001, 2005). For three-joint reaching movements of the shoulder, elbow, and wrist joints, the optimal arm movements selected by the TC were found to be highly dependent on joint viscosity. In particular, when the wrist joint viscosity was set to twice the reference value measured by Gomi and Kawato (1995), the TC reproduced the fingertip trajectories and arm postures of the measured reaching movements. However, with decreasing joint viscosity, the optimal arm movement deviated from the measured one. Consequently, when the wrist joint viscosity was similar to or lower than the reference value, the wrist joint excessively rotated, resulting in a notable deviation from the measured fingertip trajectory and arm posture. Therefore, regarding TC validity, the question arises as to whether the viscosity value of the wrist joint is approximately twice the reference value. The wrist joint viscosity value of $D_{33}$ was approximately 0.7 Nm/(rad/s) under the B10A10 condition; consequently, the estimated value for the B20A10 condition was 1.4 Nm/(rad/s). There are no reports of viscosity values exceeding 1 Nm/(rad/s) at the wrist joint, except in cases where coactivation is voluntarily increased. Therefore, the TC may be unable to reproduce three-joint reaching movements, although it can reproduce the hand trajectories of two-joint reaching movements with the shoulder and elbow joints. Consequently, it is crucial to examine three-joint arm movements with redundant degrees of freedom when evaluating the plausibility of computational models for reaching movement selection. However, the plausibility of the computational models for movement selection should be evaluated by simultaneously estimating the viscosity values of the three joints for each participant. However, notably, estimating the correct values would be extremely difficult.

Previous research has debated which computational model is more appropriate for human movement selection: a model based on kinematic constraints or one based on dynamic constraints (e.g., Soechting and Flanders 1998). In this study, however, the performances of the AJ based on a kinematic constraint and the MSC based on a dynamic constraint considering the musculoskeletal system were equivalent. Therefore, the findings of this study are inconclusive. Further probing into this issue is necessary. In addition, although this study did not address computational models that are not based on smoothness constraints, such as the minimum variance model (Harris and Wolpert 1998), a comparison with these models is important.

### Limitation of the objective function for the first optimization step of the MSC

The muscle tensions selected in the first optimization step of the MSC minimize the coactivation level of the agonist and antagonist muscles at each time. Consequently, the muscle tensions at the start and end of the movement become zero (Fig. 11), because the joint torques are zero at both ends of the movement. However, human muscles exhibit slight activation, even when the human arm is static at the initial position. This issue must be considered when evaluating the MSC. To resolve this issue, it is necessary to know all the muscle tensions of the participant’s muscles at the start of the movement. Even when measuring electromyography (EMG) for each muscle, accurately estimating muscle tension remains challenging. Herein, as described above, three types of practice and a golf waggle-like movement were performed such that the movement trial was as relaxed as possible. Therefore, it is reasonable to assume that the coactivation levels were low during the movement. Therefore, this issue is unlikely to be a crucial concern of this study. However, when using the objective function of the first optimization step, it is necessary to measure arm movements that satisfy the constraints of muscle tension and coactivation: minimum coactivation during movement and negligible muscle tension at the start and end of the movement. The MSC may be unable to explain human arm movements that do not satisfy these constraints. Therefore, it is necessary to reconsider the objective function in the first optimization step when targeting arm movements with high coactivation levels.

### Relation to the minimum motor-command change model

This section discusses the relation between the MSC and minimum motor-command-change model (MCC) proposed by Kawato (1996). The maximum muscle stress during the maximum voluntary contraction is $\sim$600 kPa (Ikai and Fukunaga 1968; Crowninshield 1978), which suggests that the maximum muscle tension is roughly proportional to the PCSA. From this perspective, it can be assumed that the muscle tension $\textrm{T}_i$ of the *i* th muscle is proportional to the product of the motor command $\alpha _i$ and the physiological cross-sectional area $\textrm{PCSA}_i$. Based on this assumption, the muscle tension is expressed as $\textrm{T}_i = k \alpha _i \textrm{PCSA}_i$, with *k* being the proportionality coefficient. Consequently, $\textrm{S}_i = k \alpha _i$ because the muscle stress $\textrm{S}_i$ is given by $\textrm{S}_i = \frac{\textrm{T}_i}{\textrm{PCSA}_i}$, indicating that the muscle stress $\textrm{S}_i$ is approximately proportional to the motor command $\alpha _i$.

The objective function $C_{MCC}$ of the MCC is expressed as follows:

11 $$\begin{aligned} C_{MCC} = \frac{1}{2}\int _0^{tf} \sum _{i=1}^m \left( \frac{d \alpha _{i}}{dt} \right) ^2 dt. \end{aligned}$$

By differentiating both sides of $\textrm{S}_i = k \alpha _i$ with respect to time, we obtain $\frac{S_i}{dt}=k\frac{d\alpha _i}{dt}$. Therefore, $C_{MSC} = k^2 C_{MCC}$. This indicates that the MSC can be regarded as an approximation of the MCC. Although it is possible to model muscles in a simple manner (Özkaya et al. 2016; Katayama and Kawato 1993), human muscles are much more complex. Consequently, evaluating the MCC is difficult. Under the above assumption, an approximate evaluation can be performed using the MSC instead of the MCC.

## Supplementary Information

Below is the link to the electronic supplementary material.

Supplementary file 1 (pdf 224 KB)

Supplementary file 2 (pdf 26 KB)

Supplementary file 3 (pdf 29 KB)

## Figures and Tables

**Fig. 13:**
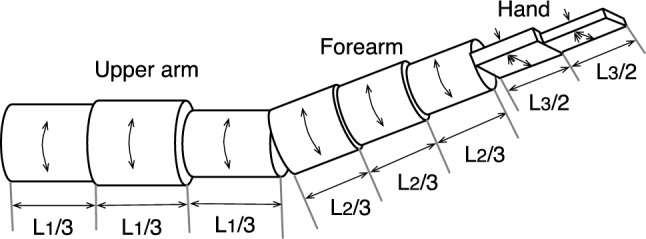
Three-joint arm model for estimating the values of the arm’s parameters

## Data Availability

The datasets and codes generated herein are available from the corresponding author upon reasonable request.

## Appendix A: Estimation of each participant’s arm parameters

To select the optimal movements of the MSC, determining the values of the physical parameters in Eq. 5 is necessary. Because these values were dependent on the participant involved, they were measured or estimated as follows: first, the link lengths of the upper arm, forearm, and hand were directly measured on each participant’s arm. The length of each link of the upper arm and forearm was determined by measuring the distance between the rotation centers of the two joints, and the length of the hand was measured from the rotation center of the wrist joint to the fingertip. Because it was difficult to directly measure the values of the remaining parameters, we modeled a three-jointed arm (Fig. 13). The upper arm and forearm were each represented by a three-cylinder model, and the circumference of each center was measured. The hand was divided into two plate parts when stretched out, and the thickness and height of the center of each part were measured. The volume of each part was calculated using the measured values. In addition to these values, the mass, center of mass, and moment of inertia of each link were estimated from the volume ratios and specific gravities of fat, muscle, and bone (Table 6). The obtained values are listed in Table 2.

**Table 6:** Volume ratios and specific gravity of fat, muscle, and bone (Inukai et al. 1971)

		Fat	Muscle	Bone
Upper arm	Male	12.4%	78.8%	8.8%
	Female	33.3%	60.7%	6.0%
Forearm	Male	11.2%	80.9%	7.9%
	Female	21.9%	70.9%	7.2%
Hand[1]	Male, Female	0.0%	50.0%	50.0%
	Specific gravity	0.9	1.04	1.2

As there are no data regarding the hand, we assumed that fat is 0% and muscle and bone are each 50%
