# Supplementary material for: Computational model to reproduce fingertip trajectories and arm postures during human three-joint arm movements: minimum muscle-stress-change model
Source: Biol Cybern. 2025 Aug 26;119(4-6):23. doi: 10.1007/s00422-025-01022-4 (PMC12380650; doi:10.1007/s00422-025-01022-4)
Supplement: Supplementary file 3 — (pdf 29 KB) [file 422_2025_1022_MOESM3_ESM.pdf]

Muscle selection

Katayama, Masazumi

Division of Human and Artificial Intelligent Systems,

Graduate School of Engineering, University of Fukui, Japan

■Caption 1:Deltoid-clavicular part (DC) 2:Pectoralis majar-clavicular part (PMC) 3:Pectoralis majar-abdominal part(PMA), 3:Coracobrachialis (Co) 4:Subscapularis (Su) 5:Deltoid-scapular part (DS) 6:Deltoid-acromial part (DA) 7:Latissimus dorsi (LD) 8:Infraspinatus (In) 9:Teres minor (TMi) 10:Teres major (TMa) 11:Brachialis head (Br) 12:Pronator teres (PT) 13:Brachioradialis (BR) 14:Triceps-medial head (MHTr) 15:Triceps-lateral head (LtHTr) 16:Anconeus (A) 17:Biceps-long head (LHBi) 18:Biceps-short head (SHBi) 19:Triceps-long head (LHTr) 20:Flexor carpi ulnaris (FCU) 21:Flexor carpi radialis (FCR) 22:Palmaris longus (PL) 23:Extensor carpi radialis brevis (ECRB) 24:Extensor carpi ulnaris (ECU) 25:Extensor carpi radialis longus (ECRL). SF, SE, EF, EE, DF, DE, WF and WE denote shoulder flexor, shoulder extensor, elbow flexor, elbow extensor, double-jointed flexor, double-jointed extensor, wrist flexor and wrist extensor, respectively. △ denotes a supplementary muscle. S11, S12, S21 and S22 were determined from Ito and Takano [2012], S31, S32, S41 and S42 were determined from Nakamura and Saito [1992]. Triangles indices supplementory muscles.

参考文献

Takashi Ito and Hiroko Takano. *Kaibogaku Kogi (Anatomy Lecture) 3th ed.* Nanzando Co., Ltd., Tokyo, 2012. in Japanese.

Ryuichi Nakamura and Hiroshi Saito. *Kiso Undogaku (Fundamental Kinesiology) 4th ed.* Ishiyaku Publishers, Inc., Tokyo, 1992. in Japanese.

Muscle	Type	Muscle selection			
		S11,S12	S21,S22	S31,S32	S41,S42
Sloulder joint					
DC	SF	○	○	○	○
PMC	SF	○	○	○	○
PMA	SF			○	○
Co	SF	○	○	○	○
Su	SF			○	○
DS	SE	○	○	○	○
DA	SE			○	○
LD	SE		△		△
In	SE			○	○
TMi	SE			○	○
TMa	SE				△
Elbow joint					
Br	EF	○	○	○	○
PT	EF	○	○		△
BR	EF		△		△
MHTr	EE	○	○	○	○
LtHTr	EE	○	○	○	○
A	EE	○	○		△
Biarticular muscles of the shoulder and elbow joints					
LHBi	DF	○	○	○	○
SHBi	DF	○	○	○	○
LHTr	DE	○	○	○	○
Wrist joint					
FCU	WF	○	○	○	○
FCR	WF	○	○	○	○
PL	WF	○	○	○	○
ECRB	WE	○	○	○	○
ECU	WE	○	○	○	○
ECRL	WE	○	○	○	○
